# Culture-negative infective endocarditis (CNIE): impact on postoperative mortality

**DOI:** 10.1515/med-2020-0193

**Published:** 2020-06-20

**Authors:** Antonio Salsano, Daniele Roberto Giacobbe, Filippo Del Puente, Roberto Natali, Ambra Miette, Sara Moscatelli, Giacomo Perocchio, Flavio Scarano, Italo Porto, Giovanni Mariscalco, Matteo Bassetti, Francesco Santini

**Affiliations:** Division of Cardiac Surgery, Ospedale Policlinico San Martino, University of Genoa, Genova, Italy; Department of Integrated Surgical and Diagnostic Sciences (DISC), University of Genoa, Genova, Italy; Clinica Malattie Infettive, Ospedale Policlinico San Martino – IRCCS, Genoa, Italy; Department of Health Sciences (DISSAL), University of Genoa, Genova, Italy; Division of Cardiology, Ospedale Policlinico San Martino, University of Genoa, Genova, Italy; Department of Internal Medicine (DIMI), University of Genoa, Genova, Italy; Department of Cardiac Surgery, Glenfield Hospital, University Hospitals of Leicester, Leicester, UK

**Keywords:** cardiopulmonary bypass, endocarditis, heart valves, inflammation

## Abstract

**Introduction:**

Poor postoperative outcomes have been reported after surgery for infective endocarditis (IE). Whether the absence of positive cultures impacts the prognosis remains a matter of discussion. The aim of this study was to evaluate the impact of negative cultures on the prognosis of surgically treated IE.

**Methods:**

This was a single-center, retrospective study. From January 2000 to June 2019, all patients who underwent valvular surgery for IE were included in the study. The primary endpoint was early postoperative mortality. A covariate balancing propensity score was developed to minimize the differences between the culture-positive IE (CPIE) and culture-negative IE (CNIE) cohorts. Using the estimated propensity scores as weights, an inverse probability treatment weighting (IPTW) model was built to generate a weighted cohort. Then, to adjust for confounding related to CPIE and CNIE, a doubly robust method that combines regression model with IPTW by propensity score was adopted to estimate the causal effect of the exposure on the outcome.

**Results:**

During the study period, 327 consecutive patients underwent valvular repair/replacement with the use of cardiopulmonary bypass and cardioplegic cardiac arrest for IE. Their mean age was 61.4 ± 15.4 years, and 246 were males (75.2%). Native valve IE and prosthetic valve IE accounted for 87.5% and 12.5% of cases, respectively. Aortic (182/327, 55.7%) and mitral valves (166/327, 50.8%) were mostly involved; 20.5% of isolated mitral valve diseases were repaired (22/107 patients). The tricuspid valve was involved in 10 patients (3.3%), and the pulmonary valve in 1 patient (<1%). Fifty-nine patients had multiple-valve disease (18.0%). Blood cultures were negative in 136/327 (41.6 %). A higher postoperative mortality was registered in CNIE than in CPIE patients (19% vs 9%, respectively, *p* = 0.01). The doubly robust analysis after IPTW by propensity score showed CNIE to be associated with early postoperative mortality (odds ratio 2.10; 95% CI, 1.04–4.26, *p* = 0.04).

**Conclusions:**

In our cohort, CNIE was associated with a higher early postoperative mortality in surgically treated IE patients after dedicated adjustment for confounding. In this perspective, any effort to improve preoperative microbiological diagnosis, thus allowing targeted therapeutic initiatives, might lead to overall better postoperative outcomes in surgically treated IE.

## 1 Introduction

Poor postoperative outcomes have been reported after surgery for infective endocarditis (IE) [1]. Treatment of IE is based on antibiotic therapy combined with an aggressive surgical debridement of infected tissue whenever indicated.

Failure to culture the causative agent (e.g., because of antimicrobial treatment prior to blood culture and/or infection with hardly detectable pathogens) has been associated with increased mortality in IE patients, both in medically and in surgically treated patients, although only a few analyses that clearly separate medical and surgical cohorts have been conducted [2,3,4,5].

In the present study, we assessed the impact of negative blood cultures on the outcome of surgically treated IE patients over a 20-year period.

## 2 Methods

### 2.1 Study design

Demographic and clinical data of patients who underwent valve repair/replacement with the use of cardiopulmonary bypass (CPB) and cardioplegic cardiac arrest for IE at the Ospedale Policlinico San Martino – IRCCS (a 1,200-bed teaching hospital in Genoa, Italy) from January 2000 to June 2019 were retrospectively retrieved from a prospectively collected institutional database. The study involved the analysis of existing anonymized clinical and laboratory data. An informed consent for the use of anonymized data for scientific purposes is signed by all patients admitted to Ospedale Policlinico San Martino – IRCCS and is included in scientific databases for subsequent data analysis. All data were stored according to Personal Data Protection Act.

### 2.2 Study endpoints

The primary binary endpoint was early postoperative mortality (defined as death during the hospital stay or within 30 days after surgery). Secondary binary endpoints were ICU stay, hospital stay, mechanical ventilation, respiratory failure, sepsis, reoperation for bleeding/tamponade, reoperation for sternal wound infection, stroke, acute kidney injury, new onset of atrioventricular block, atrial fibrillation, multi-organ failure, and long-term mortality (as a time-to-event endpoint).

### 2.3 Definitions and procedures

The presence of IE was defined according to the modified Duke criteria [6,7]. All the other patients who did not fulfill the modified Duke criteria preoperatively were retrospectively included if cultures of vegetation and/or abscess and/or tissue histology collected at the time of surgery turned out to be positive, thereby confirming the presence of IE.

Culture-negative infective endocarditis (CNIE) was defined as the absence of microorganism identification either in blood cultures or in tissue cultures from the excised valve or vegetation.

Patient’s comorbidities and postoperative complications were defined according to the joint AATS/EACTS/STS Guidelines for Reporting Mortality and Morbidity After Cardiac Valve Interventions in adult cardiac surgery [8].

Urgent–emergent surgery priorities were defined as surgery performed immediately or within the current admission for medical reasons according to the EuroSCORE II definitions [9].

Myocardial protection was achieved by means of anterograde administration of warm blood cardioplegia associated with topical ventricular cooling or hypothermic (31°C) Custodiol HTK (histidine–tryptophan–ketoglutarate) solution.

The duration of antimicrobial therapy was in line with ESC guidelines [10].

### 2.4 Data collected for the analysis

The following baseline variables were collected: gender; age (collected as a continuous variable); hypertension; diabetes (any preoperative diagnosis of diabetes mellitus requiring treatment [diet, oral drugs, and/or insulin therapy]); obesity (body mass index [BMI] > 30); chronic obstructive pulmonary disease (COPD, defined as long-term use of bronchodilators or steroids for lung disease); stroke (any preoperative focal or global neurological syndrome caused by ischemia or hemorrhage not resolving within 24 h); previous cardiac surgery; peripheral vascular disease (one or more of the following: claudication, carotid occlusion or >50% stenosis, amputation for arterial disease, previous or planned intervention on the abdominal aorta, limb arteries, or carotids); preoperative insertion of intra-aortic balloon pump (IABP); chronic kidney disease (baseline serum creatinine > 200 µmol/L); solid-organ cancer; preoperative mechanical ventilation; myocardial infarction <3 months; New York Heart Association (NYHA) class; European System for Cardiac Operative Risk Evaluation II (EuroSCORE II, calculated retrospectively using the online calculator available at http://www.euroscore.org/calc.html) [9]; urgent and emergent priorities defined according to EuroSCORE II definitions; endocarditis characteristics (native [NVE] or prosthetic valve endocarditis [PVE]; cerebrovascular embolic events in the setting of IE; causative organisms (identified by standard microbiological procedures in positive blood or valvular tissue cultures); 2D echocardiographic data (the presence of vegetation, abscess, paravalvular leak, LVEF, valve diseases, pulmonary hypertension); atrioventricular block on the electrocardiogram (ECG); and operative findings.

The collected postoperative complications during the hospital stay after surgery included the following: resternotomy for bleeding; mechanical ventilation (hours); intensive care unit (ICU) stay (days); hospital stay (days); acute kidney injury (a peak serum creatinine ≥50% above baseline within 5 days postoperatively); postoperative stroke, atrial fibrillation; new onset of atrioventricular block; sternal wound infection requiring resternotomy; respiratory insufficiency (any of hypoxemic respiratory failure with a PaO_2_/FiO_2_ ratio less than 300 while on supplemental oxygen or acute hypercapnic respiratory failure, defined as a significantly elevated PaCO_2_ to 50 mmHg or more and a pH less than 7.35); sepsis (according to international definitions) [11]; multi-organ failure (defined as multiple-organ dysfunction within the context of systemic inflammatory response syndrome, SIRS); and reoperation for bleeding/tamponade.

### 2.5 Statistical analysis

For descriptive purposes, categorical data were presented as frequencies and percentages and compared using the Chi-square test or Fisher’s exact test where appropriate. Continuous variables were expressed as mean ± standard deviation (SD) or median and interquartile range [IQR] and compared using two-tailed *t*-test or Mann–Whitney test.

The primary study analysis consisted in the assessment of factors associated with early postoperative mortality, with special focus on the prognostic impact of CNIE. To adjust for confounding, a doubly robust method that combines regression model with inverse probability treatment weighting (IPTW) by propensity score was adopted to estimate the causal effect of the exposure on mortality [12]. In more detail, a covariate balancing propensity score (CBPS) was developed to minimize the differences between the culture-positive infective endocarditis (CPIE) and CNIE cohorts [13]. In our study, a total of 35 covariates including preoperative baseline and operative characteristics were used in the model. The full list of these covariates is given in Table 1 and Supplementary Table 1. Using the estimated propensity scores as weights, an inverse probability weighting (IPW) model was used to generate a weighted cohort [14]. C-statistics were calculated to ascertain the validity of the propensity score. The effect of CNIE on the outcome was also adjusted for the EuroSCORE II. The discriminatory ability of the regression model was assessed by the receiver operating characteristics (ROC) curve test. The improvement in the predictive accuracy of the regression model before and after the addition of CNIE was estimated by calculating the net reclassification index (NRI) and integrated discrimination improvement (IDI) [15].

**Table 1:** Preoperative clinical and operative characteristics of patients with CPIE versus CNIE in the overall series

Variable^a^	Overall series		
	CPIE *N* = 191	CNIE *N* = 136	*P* value
Female gender	47 (24.6)	34 (25.0)	1.00
Age	64.00 [47.50, 72.00]	66.50 [53.75, 74.25]	0.02
Hypertension	85 (44.5)	55 (40.4)	0.54
Diabetes	27 (14.1)	19 (14.0)	1.00
BMI > 30	26 (13.6)	31 (22.8)	0.05
COPD	15 (7.9)	14 (10.3)	0.57
Stroke	22 (11.5)	13 (9.6)	0.70
Previous cardiac surgery	35 (18.3)	34 (25.0)	0.19
Peripheral vascular disease	18 (9.4)	11 (8.1)	0.83
Preoperative IABP	9 (4.7)	10 (7.4)	0.44
Chronic kidney disease	32 (16.8)	22 (16.2)	1.00
Solid-organ cancer	5 (2.6)	5 (3.7)	0.82
Cirrhosis	4 (2.1)	1 (0.7)	0.60
Preoperative mechanical ventilation	9 (4.7)	10 (7.4)	0.44
Myocardial infarction < 3 months	2 (1.0)	3 (2.2)	0.70
Unstable angina	2 (1.0)	5 (3.7)	0.22
NYHA I–II	143 (74.9)	90 (66.2)	0.11
NYHA III–IV	48 (25.1)	46 (33.8)	0.11
EuroSCORE II	2.94 [1.60, 5.64]	3.12 [1.60, 11.21]	0.33
Endocarditis characteristics			
NVE	162 (84.8)	124 (91.2)	0.12
PVE	29 (15.2)	12 (8.8)	0.12
Aortic valve involvement	109 (57.1)	73 (53.7)	0.62
Mitral valve involvement	98 (51.3)	68 (50.0)	0.90
Multiple-valve disease	35 (18.3)	24 (17.6)	0.88
Embolic event	8 (4.2)	2 (1.5)	0.28
Echocardiogram and electrocardiogram findings			
Vegetation >1 cm	29 (15.2)	14 (10.3)	0.26
Abscess	10 (5.2)	4 (2.9)	0.46
Paravalvular leak	15 (7.9)	6 (4.4)	0.31
Severe stenosis	10 (5.2)	9 (6.6)	0.77
Severe regurgitation	186 (97.4)	131 (96.3)	0.82
LVEF, %	55.00 [50.00, 60.00]	55.00 [45.00, 57.00]	0.15
PAPS > 60 mmHg	18 (9.4)	19 (14.0)	0.27
Atrioventricular block	7 (3.7)	12 (8.8)	0.08
Surgical procedure			
Single-valve procedure	156	112	0.88
Multiple-valve procedure	35 (18.3)	24 (17.6)	0.88
Concomitant CABG	7 (3.7)	4 (2.9)	0.96
Concomitant procedure on thoracic (ascending) aorta	13 (6.8)	8 (5.9)	0.92
Urgent–emergent	84 (44.0)	53 (39.0)	0.43
CPBT	118.00 [86.00, 154.50]	116.00 [86.25, 150.50]	0.80
XCT	86.00 [60.00, 114.00]	80.00 [61.00, 107.25]	0.68

BMI: body mass index; CABG: coronary artery bypass grafting; CNIE, culture-negative infective endocarditis; COPD: chronic obstructive pulmonary disease; CPBT: cardiopulmonary bypass time; IABP: intra-aortic balloon pump; CPIE, culture-positive infective endocarditis; LVEF: left ventricular ejection fraction; NVE: native valve endocarditis; NYHA: New York Heart Association; PAPS: pulmonary artery systolic pressure; PVE: prosthetic valve endocarditis; XCT: aortic cross-clamp time.

^a^Continuous data are presented as median [interquartile range], and categorical variables as number (percent).

The same doubly robust method was used to address the impact of CNIE on the following secondary outcomes: ICU stay, hospital stay, mechanical ventilation, respiratory failure, sepsis, reoperation for bleeding/tamponade, reoperation for sternal wound infection, stroke, acute kidney injury, new onset of atrioventricular block, atrial fibrillation, and multi-organ failure.

The long-term mortality in CNIE and CPIE patients was assessed using the Kaplan–Meier curve and IPTW by propensity score to build adjusted survival curves [16]. Statistical analyses were performed using cobalt and PredictABEL packages of R software (version 3.5.1; R Foundation for Statistical Computing, Vienna, Austria) [17,18].

## 3 Results

Overall, 327 consecutive patients underwent valve repair/replacement with the use of CPB and cardioplegic cardiac arrest for IE from January 2000 to June 2019. Their mean age was 61.4 ± 15.4 years, and 246 were males (75.2%). NVE and PVE accounted for 87.5% and 12.5% of cases, respectively. Aortic (182/327, 55.7%) and mitral valves (166/327, 50.8%) were mostly involved; 20.5% of isolated mitral valve diseases were repaired (22/107 patients). The tricuspid valve was involved in 10 patients (3.3%), and the pulmonary valve in 1 patient (<1%). Fifty-nine patients had multiple-valve disease (18.0%), and sixty-nine patients had previous cardiac surgery (21.1%), including sixty-eight valvular procedures and one coronary artery bypass grafting. Median EuroSCORE II was 3.01 with IQR 1.60–7.59. Demographic and clinical characteristics of the included patients are shown in Table 1 and Supplementary Table 1.

CPIE (191/327, 58,4%) occurred more frequently than CNIE (136/327, 41,6%). Staphylococci (73/191, 38.2%) and streptococci (65/191, 34%) were the most commonly involved bacteria in the CPIE group. Details about the causative agents are reported in Table 2. A minimum number of three blood cultures were performed in all CNIE patients.

**Table 2:** Causative agents of CPIE^a^

Causative agent	*N* (%)
*Staphylococcus aureus*	57 (30.0)
Coagulase-negative staphylococci	
*Staphylococcus epidermidis*	18 (9.4)
*Staphylococcus hominis*	4 (2.1)
*Staphylococcus capitis*	2 (1.0)
*Staphylococcus haemolyticus*	2 (1.0)
Streptococci	
Viridans group streptococci^b^	37 (19.4)
*Streptococcus bovis* group	16 (8.4)
*Streptococcus pyogenes*	1 (0.5)
*Streptococcus pneumoniae*	1 (0.5)
*Enterococcus* spp.	
*Enterococcus faecalis*	39 (20.4)
*Enterococcus faecium*	2 (1.0)
Nutritionally variant streptococci	
*Abiotrophia defectiva*	1 (0.5)
*Granulicatella adiacens*	1 (0.5)
Gram-negative bacteria	6 (3.1)
*Candida* spp.	3 (1.6)
Other organisms	1 (0.5)
Total	191 (100)

^a^All etiological diagnoses of surgically treated IE were made through cultures. No diagnosis was made through molecular methods, and there were no serological diagnoses of *Coxiella burnetii* endocarditis in operated patients.

^b^With the exception of *Streptococcus bovis* group and *Streptococcus pneumoniae*.

Briefly, the CNIE group was older (CNIE median 66.5 years [IQR, 53.8–74.3], versus CPIE median 64.0 years, [IQR, 47.0–72.0], *P* = 0.02) and more obese (31/136 CNIE patients versus 26/191 CPIE patients, *P* = 0.05). A linear decrease in the yearly proportion of CNIE/operated IE was observed during the study period (Chi-square test for trend in proportion = 30.413, *p* < 0.001), reaching a mean value of 26% during the last five study years.

The overall in-hospital mortality was 13.5% (44/327 patients). Early mortality rates varied during the study period without significant changes over years (Chi-squared test for independence = 1.969, *p* = 0.161; Chi-squared test for trend in proportions = 0.00086, df = 1, *p* value = 0.977; Supplementary Figure 1). CNIE had a higher early postoperative mortality compared to CPIE (26/136 patients [19.1%] vs 18/191 patients [9.4%], *p* = 0.01). Complete descriptive comparisons of primary and secondary outcomes between CNIE and CPIE patients are summarized in Table 3.

**Table 3:** Early outcomes between patients with CPIE and CNIE and the doubly robust matching estimators for confounding adjustment

Variables^a^	Overall series			Doubly robust adjustment^b^		
	CPIE *N* = 191	CNIE *N* = 136	*P* value	Odds ratio	95% CI	*P* value
Primary endpoint						
In-hospital/30-day mortality	18 (9.4)	26 (19.1)	0.02	2.10	1.03–4.26	0.04
Secondary endpoints						
ICU stay, days	2.00 [1.00, 4.00]	2.00 [1.00, 5.00]	0.40	−0.11^c^	0.12^c^	0.36^c^
Hospital stay, days	11.00 [7.00, 18.00]	7.00 [4.00, 11.50]	<0.001	−4.32^c^	1.37^c^	0.002^c^
Mechanical ventilation, hours	12.00 [11.00, 26.00]	15.00 [9.75, 36.00]	0.79	34.67^c^	35.49^c^	0.33^c^
Respiratory failure	16 (8.4)	9 (6.6)	0.71	0.76	0.30–1.96	0.56
Sepsis	14 (7.3)	14 (10.3)	0.46	1.44	0.63–3.30	0.38
Reoperation for bleeding/tamponade	0	0	N.A.	N.A.	N.A.	N.A.
Reoperation for sternal wound infection	7 (3.7)	4 (2.9)	0.96	0.46	0.12–1.73	0.25
Stroke	2 (1.0)	2 (1.5)	1.00	0.81	0.11–5.83	0.83
Acute kidney injury	16 (8.4)	8 (5.9)	0.52	0.69	0.28–1.83	0.46
Atrioventricular block	9 (4.7)	3 (2.2)	0.37	0.61	0.16–2.39	0.49
Atrial fibrillation	44 (23.0)	24 (17.6)	0.30	0.79	0.43–1.44	0.44
Multi-organ failure	3 (1.6)	7 (5.1)	0.13	4.47	1.07–18.65	0.04

CI, confidence interval; CNIE, culture-negative infective endocarditis; CPIE, culture-positive infective endocarditis; ICU, intensive care unit; N.A., not applicable.

^a^Continuous data are presented as median [interquartile range], and categorical variables as number (percent).

^b^Reference for the events: CPIE cohort.

^c^Linear regression has been expressed as standard regression coefficient, standard error, and *P* value.

**Figure 1:**
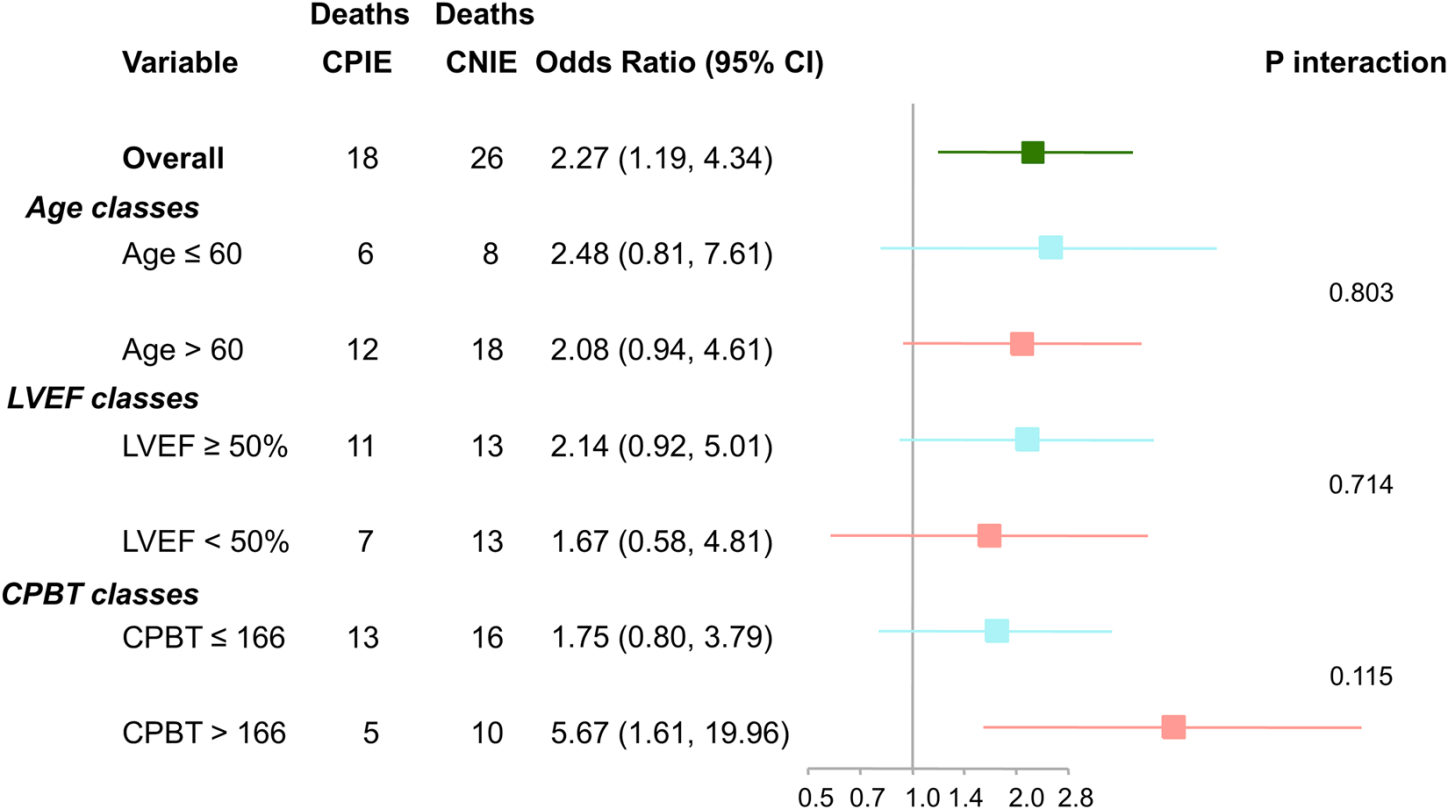
Subgroup analysis for in-hospital mortality with variables’ interactions.

As shown in Supplementary Table 1 and Supplementary Figures 2 and 3, all the covariates of the weighted cohort resulted in balance between groups. The C-statistics of the propensity score was 0.724. Under the doubly robust estimation framework, the regression models demonstrated that CNIE was associated with a significantly higher risk of early postoperative mortality (OR 2.10; 95% CI, 1.03–4.26; *P* = 0.04). Sensitivity analyses and variable interactions that considered age, preoperative left ventricular ejection fraction, and CPBT showed that CNIE impacted early postoperative mortality in the absence of significant interactions with these risk factors (Figure 1) [19]. Of note, adding the variable “CNIE” to the EuroSCORE II for the prediction of early postoperative mortality resulted in a larger AUC (0.707, 95% CI 0.623–0.792) than the EuroSCORE II (AUC 0.684, 95% CI 0.596–0.772), but the difference failed to reach statistical significance (*p* = 0.493). However, the IDI was 1.5% (*p* = 0.05) and NRI was 40% (*p* = 0.01) (Table 4).

**Table 4:** Reclassification table comparing early mortality risk models without and with CNIE

	Reclassified predicted risk (with CNIE)						% (*N*) of subjects reclassified with		
Predicted risk (without CNIE)	<10%	10 to <20%	20 to <30%	30 to <40%	40 to <50%	≥50%	Increased risk (%)	Decreased risk (%)	Net correctly reclassified (%)
Survived patients (*N* = 283)									
<10%	47	35	0	0	0	0	16.96%	37.81%	20.85%
10 to <20%	100	70	11	0	0	0	(48)	(107)	
20 to <30%	0	4	6	1	0	0			
30 to <40%	0	0	1	2	1	0			
40 to <50%	0	0	0	2	1	0			
≥50%	0	0	0	0	0	2			
Updated model with in-hospital/30-day mortality (*N* = 327)									
<10%	49	38	0	0	0	0	17.13%	36.70%	19.57%
10 to <20%	109	84	14	0	0	0	(56)	(120)	
20 to <30%	0	7	9	3	0	0			
30 to <40%	0	0	2	4	1	0			
40 to <50%	0	0	0	2	1	0			
≥50%	0	0	0	0	0	4			
Net reclassification improvement (95% CI)							0.40 (0.09–0.72), *P* = 0.01		

With regard to secondary outcomes, CNIE was associated with multi-organ failure (OR 4.47; 95% CI, 1.07–18.65; *P* = 0.04), whereas CPIE was associated with a longer hospital stay (linear regression estimate −4.32; standard error 1.37; *P* = 0.002). No significant differences were observed between the two groups with regard to the other secondary outcomes assessed under the doubly robust estimation framework.

**Figure 2:**
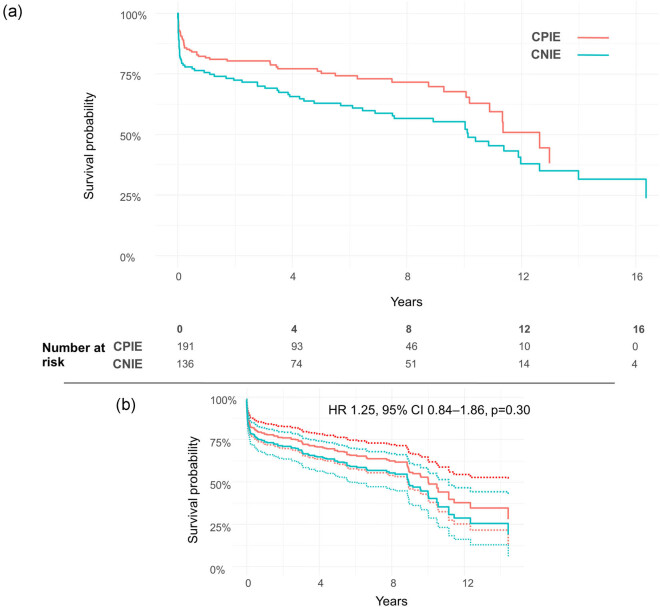
(a) Kaplan–Meier survival curves stratified for CPIE and CNIE; (b) adjusted survival curves.

The mean follow-up was 61.3 ± 56.4 months. Actuarial survival at 1, 5, and 10 years were 81 ± 3%, 76 ± 3%, 67 ± 5% and 76 ± 4%, 63 ± 4%, 52 ± 5% for patients with CPIE and CNIE, respectively. The two curves demonstrated to be significantly different at log-rank test (*p* = 0.025, Figure 2a); nevertheless, adjusted survival curves using IPTW by propensity score failed to show differences in the long-term survival for CNIE cohort (HR 1.25, 95% CI 0.84–1.86, *p* = 0.30, Figure 2b).

## 4 Discussion

CNIE is a difficult-to-diagnose condition encompassing infective and non-infective etiologies. Negative cultures in case of IE are mostly caused by sterilization of blood cultures due to prior antibacterial treatment, fastidious microorganisms such as HACEK bacteria or nutritionally variant streptococci, which may require prolonged culture incubation for identification, or intracellular bacteria that cannot be routinely cultured in blood with the currently used techniques in most non-research laboratories [20,21].

Empirical antibiotic therapy is the main cause of negative culture in CNIE patients, being responsible for 50% of CNIE cases, followed by the presence of fastidious bacteria which accounts for 15–20% of CNIE cases [22]. Non-IE is very rare and includes marantic endocarditis or endocarditis related to systemic diseases, such as lupus and Behçet [20]. In our series, 41.6% of patients had CNIE, compared with 15–40% in previous reports [1,2,3,4,23,24]. Nonetheless, it should be noted that the yearly prevalence of CNIE significantly decreased over the study period, being <30% during the last study years.

According to the currently available literature, the prognostic impact of CNIE is controversial. Phua et al. reported comparable mortality rates and in-hospital course for both CNIE and CPIE patients [25]. The study by Zamorano et al. showed that CNIE had a higher frequency of heart failure and valve rupture or perforation, as well as a tendency to more frequent need of surgical repair, although mortality was not different than CPIE [2]. Conversely, Diez-Villanueva et al. found CNIE to be an independent predictor of early mortality and postoperative heart failure [5]. As specifically regards surgically treated IE, in the study of Murashita et al., CNIE was an independent predictor of poor late survival and increased rate of postoperative adverse events, but it was not associated with early mortality [3]. In Di Mauro et al.’s study, CNIE was not associated with early postoperative mortality [23]. On the contrary, in our study early postoperative mortality was higher in CNIE patients than in CPIE patients (19.1% versus 9.4%, respectively), with CNIE significantly and unfavorably impacting the outcome in a regression model built to minimize the possible confounding effect of the differences between CNIE and CPIE patients, in order to better assess the specific prognostic effect of CNIE. Speculatively, this is in line with the hypothesis that the absence of positive microbiological specimens could hamper the effectiveness of therapy and the ability to reduce systemic inflammation in operated patients, at least in some cases of CNIE. The impact of CNIE on early postoperative mortality could also be the consequence of the recirculation of shed pericardial and mediastinal blood, possibly contaminated by infected valve tissue during surgery, combined with the acute systemic inflammatory response that might unfavorably influence the perioperative course [26].

Notably, negative blood cultures are not included in prognostic scores designed for patients undergoing surgery, even in the most recent risk scores specifically conceived for IE [23,27,28,29,30]. We therefore added the variable “CNIE” to the EuroSCORE II to test the prediction of in-hospital/30-day mortality [19]. It is noteworthy that this resulted in a prognostic performance improvement, although this result needs further validation. Should this be confirmed, CNIE would be eventually recognized as a potential incremental risk factor for early postoperative mortality, thus contributing to improve dedicated risk scores efficacy.

Our study has the following important limitations. This is an observational retrospective study with possible information and selection biases, although a propensity weighting was applied to reduce confounding. In addition, we were retrospectively unable to verify the contribution of possible variations in microbiological diagnostic procedures and antibiotic therapy algorithms during the study period, which may have affected the prognosis. Finally, surgical procedures were performed over 18 years by different surgical teams, although surgical techniques have not been substantially changed over time.

## 5 Conclusion

In our cohort, CNIE was associated with higher early postoperative mortality in surgically treated IE patients after dedicated adjustment for confounding, thus emerging as a specific additional risk factor. The addition of CNIE to classical prognostic scores, provided our results are validated in external cohorts, might improve predictive efficacy. Efforts to improve preoperative microbiological diagnosis might lead to overall better postoperative outcomes in surgically treated IE.
